# Mobile phones of paediatric hospital staff are never cleaned and commonly used in toilets with implications for healthcare nosocomial diseases

**DOI:** 10.1038/s41598-021-92360-3

**Published:** 2021-06-21

**Authors:** Matthew Olsen, Anna Lohning, Mariana Campos, Peter Jones, Simon McKirdy, Rashed Alghafri, Lotti Tajouri

**Affiliations:** 1 Faculty of Health Sciences and Medicine, Bond University, Robina, QLD Australia; 2 Dubai Police Scientists Council, Dubai Police, Dubai, United Arab Emirates; 3 Harry Butler Institute, Murdoch University, Murdoch, WA 6150 Australia; 4 Dubai Future Council on Community Security, Dubai, United Arab Emirates; 5 Genomics and Molecular Biology, Bond University, Gold Coast, QLD 4229 Australia

**Keywords:** Health care, Risk factors

## Abstract

An ever-increasing number of medical staff use mobile phones as a work aid, yet this may pose nosocomial diseases. To assess and report via a survey the handling practices and the use of phones by paediatric wards healthcare workers. 165 paediatric healthcare workers and staff filled in a questionnaire consisting of 14 questions (including categorical, ordinal and numerical data). Analysis of categorical data used non-parametric techniques such as the Chi-squared test. Although 98% of respondents (165 in total) report that their phones may be contaminated, 56% have never cleaned their devices. Of the respondents that clean their devices, 10% (17/165) had done so with alcohol swabs or disinfectant within that day or week; and an additional 12% respondents (20/165) within that month. Of concern, 52% (86/165) of the respondents use their phones in the bathroom, emphasising the unhygienic environments in which mobile phones/smartphones are constantly used. Disinfecting phones is a practice that only a minority of healthcare workers undertake appropriately. Mobile phones, present in billions globally, are therefore Trojan Horses if contaminated with microbes and potentially contributing to the spread and propagation of micro-organisms as per the rapid spread of SARS-CoV-2 virus in the world.

## Introduction

Two-thirds of the world’s population has a mobile phone with roughly three-quarters of all mobile handsets being smartphone devices^1^. With the extensive availability of mobile devices and smartphones throughout the world, the healthcare sector has adapted to using these devices as a work aid in an effort to increase quality of care. Most doctors, nurses and healthcare staff of all levels of seniority regularly use either their personal mobile phones/smartphones or hospital working phones to communicate and provide efficient medical advice across departments in healthcare settings^2^.

A multitude of medical applications and software are available for use on mobile devices and are frequently used and encouraged in hospitals in an effort to increase access to point-of-care tools, to provide greater clinical decision-making and to achieve superior patient outcome^3^. On average, it is estimated that individuals spend 3 h and 37 min using their mobile devices per day^1^. A 2014 survey consisting of 109 doctors outlined that 91% of respondents owned a smartphone and 88% used their mobile devices regularly in the clinical setting^2^. Similar results were seen in a 2013 Australian study outlining 87% of healthcare professionals used their mobile phones during clinical practice^4^.

Recently, it has been demonstrated that mobile phones are contaminated platforms with a large spectrum of microorganisms, with an average contaminated rate of 68%^5^. Additionally, the phones of healthcare workers demonstrated a high occurrence of bacteria with antimicrobial resistance^5^ emphasising an avenue for mobile phones as “Trojan Horses” to contribute to the spread of nosocomial diseases in healthcare settings.

The United States Centre for Disease Control and Prevention (CDC) estimate approximately 80% of all infectious disease is transmitted via contact with hands^6^. Moreover, the COVID-19 pandemic has emphasised the necessity of proper hand hygiene and behavioural regulation to mitigate the transmission of this infectious disease. Whilst clear guidelines for hand washing are already implemented and outlined by the CDC, there are currently very limited policies for phones and no regulations for highly touched mobile phones in healthcare settings with few studies exploring the attitudes of staff towards smartphone regulation. Devices are very rarely decontaminated, and phone hygiene is often overlooked^7^.

A study by Brady et al., 2011 utilised a well-defined but limited questionnaire as a means of categorising individual’s smartphone habits and opinions about the microorganisms detected on the devices. 102 (70.3%) respondents were aware that their device could harbour harmful bacteria and 52 (50.9%) indicated that they have never cleaned their phone outside of the hospital environment^8^. Additionally, a 2020 Italian study undertaken by Cicciarella Modica et al. utilised a questionnaire to assess a limited number of participants (n = 108) specifically oriented to students for their mobile phone habits in a healthcare setting. From this study, 93% of students used mobile phones in hospitals, 72% used their devices without gloves and 33% frequently clean their mobile phones^9^.

In order to address the limitations of previous studies this research consisted of a survey of medical staff and non-medical staff, from four wards. The wider scope of our investigation ensured the survey was able to collect comprehensive information on the usage of mobile phones in healthcare settings and to feature habits surrounding the use of such devices at work.

This study focused on gathering demographic and quantitative data, with particular attention as to whether an individual uses their mobile device in the bathroom, whether they believe that mobile phones harbour micro-organisms and whether participants take any action to keep their devices clean.

The questionnaire was paired with swab samples of the phones, which our team analysed for the presence of a wide spectrum of viable microbes (manuscript in submission).

## Methods

This study was conducted in an acute paediatric healthcare setting consisting of 165 working staff members at the Gold Coast University Hospital, Australia. Data was collected through a self-completion questionnaire (Appendix), consisting of 14 questions and 7 sub questions relating to mobile phone usage and hygiene habits. The anonymous questionnaire took approximately 5–10 min to complete and was conducted across 4 different paediatric wards: General Paediatrics (GP), Paediatric Intensive Care Unit (PICU), Neonatal Intensive Care Unit (NICU) and Paediatric Emergency Department (PED). A participant information sheet was provided, detailing the project and ensuring respondents that personal information would not be collected to ensure anonymity. Subsequently informed consent was provided by agreeing to participate on the day.

Recruitment was based on convenience sample (December 2018-December 2019). Staff at the Gold Coast University Hospital were invited to participate both before and during their respective shifts. Strategies were implemented to limit opportunities for participant behaviour changes at the time of the survey by preventing advance notice of the research to participants.

Participants consisted of medical staff (including doctors, ward nurses, nurse manager, assistant in nursing, nurse practitioner, ward pharmacist and outpatient clinic staff) and non-medical staff (including facilities staff and working individuals who did not specify their occupation).

Most questions (Appendix) consisted of tick-box responses with binary yes/no answers, for example, ‘have you recently used your phone/device while using the toilet/bathroom?’ Whilst sub-questions provided a range of potential answers, for example, “if yes, for which purpose would you be most likely to be using on your device at this time? with potential responses being work/social media/personal phone calls/mobile gaming/other.

### Ethics

This research was approved by the Gold Coast University Hospital Human Research Ethics Committee with Site Specific approval (GC HREA 46569) as well as Bond University Human Research Ethics Committee approval (16004).

### Statistical analysis

Associations between participant demographics and survey responses were analysed using Chi-Square Test of independence. Frequency tables were analysed to compare, for example, participant occupation, age and gender against mobile phone use in the bathroom, frequency of mobile phone cleaning and method used to clean mobile phones. P values were presented without adjustment for statistical analysis and statistical significance was determined by P = 0.05.

## Results

### Participant demographics

In total, there were 165 healthcare workers who participated in this survey (Table 1). Of these, 45% were working in the General paediatrics, 23% were from the PICU, 15% from NICU and 15% from PED.

**Table 1:** Total count of participant occupations.

Occupation	Totals	%
**Medical staff**		
Doctors	54	33
Ward nurse, nurse manager, assistant in nursing and nurse practitioner	83	50
Students, including medical students and nursing students	18	11
Ward pharmacist	4	2.4
Outpatient clinic staff	1	0.6
**Non-medical staff**		
Facilities staff (cleaners)	2	1.2
Unspecified	3	1.8
Grand total (N)	165	100

### Mobile phone use and characteristics

Besides personal use, 80% (n = 132) of respondents claimed to use their personal mobile phones for work-based activities and 87% (n = 143) believed that their mobile phones were essential tools for their job. At the time of the survey, 73% (121/165) and 27% (44/165) of respondents utilised large and small screens smartphone devices respectively and 58% (95/165) of participants used a phone cover. In terms of age of the appliance, 13% of respondents utilised devices between 0–6 months in age, 22% between 6–12 months, 58% greater than 12 months, and 7% of respondents did not specify the age of their phones.

### Mobile phone health and hygiene habits

No participants were taking antibiotics at the time of the survey. Nonetheless, approximately 12% of ward nurses and 22% of doctors reported to be feeling mildly unwell. Of note, regarding hand washing, 84% of participants utilised water and soap whereas 15% did not specify their hand washing method of choice, and finally 1 participant utilised hand sanitizer.

**Figure 1:**
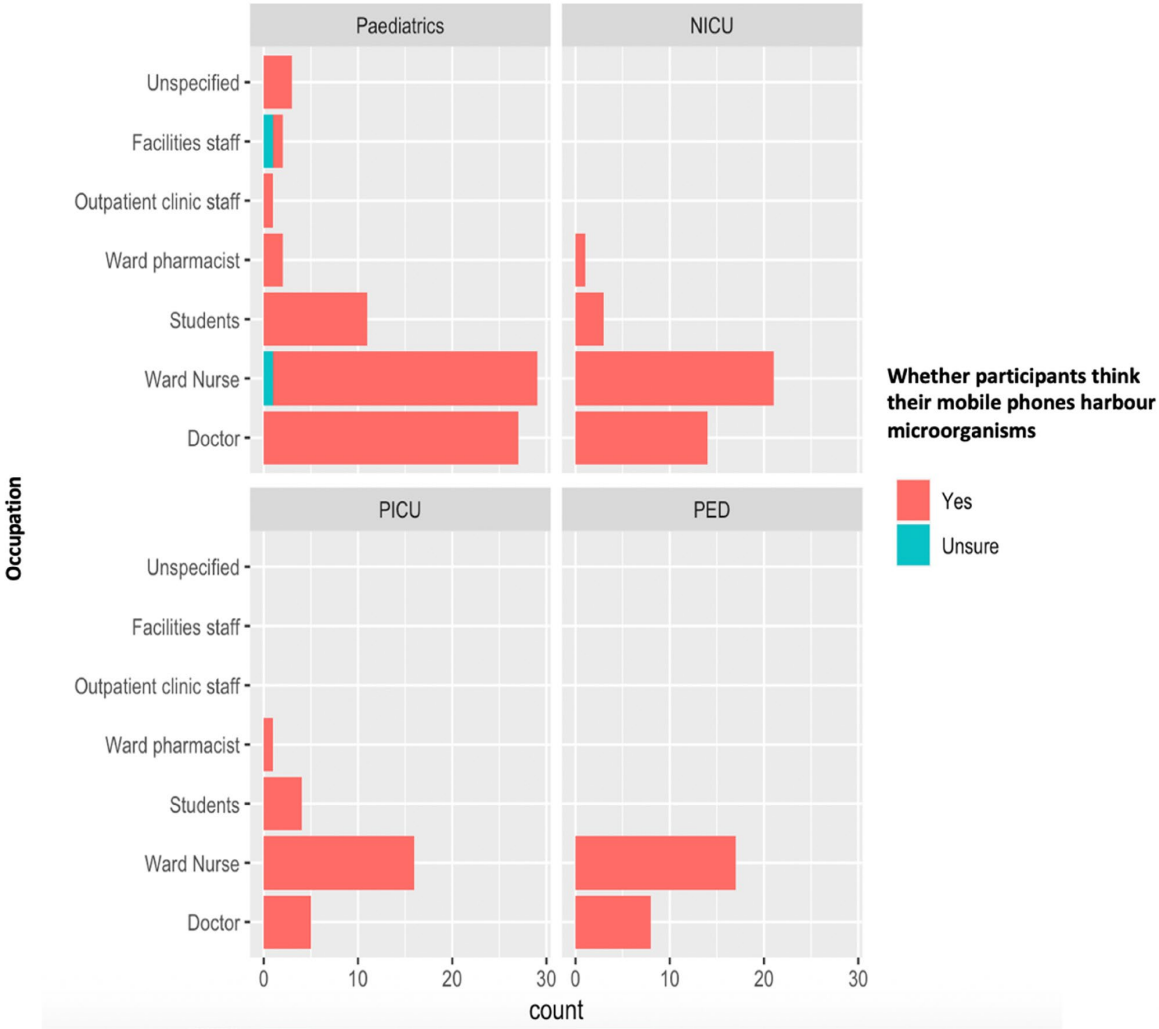
Mobile phone contamination awareness and staff role across four different hospital wards (General Paediatrics, Neonatal Intensive Care Unit, Paediatric Intensive Care Unit, Paediatric Emergency Department).

When exploring the awareness of mobile phone contamination, 98.7% of participants thought that their phones could carry microorganisms (Fig. 1).

**Figure 2:**
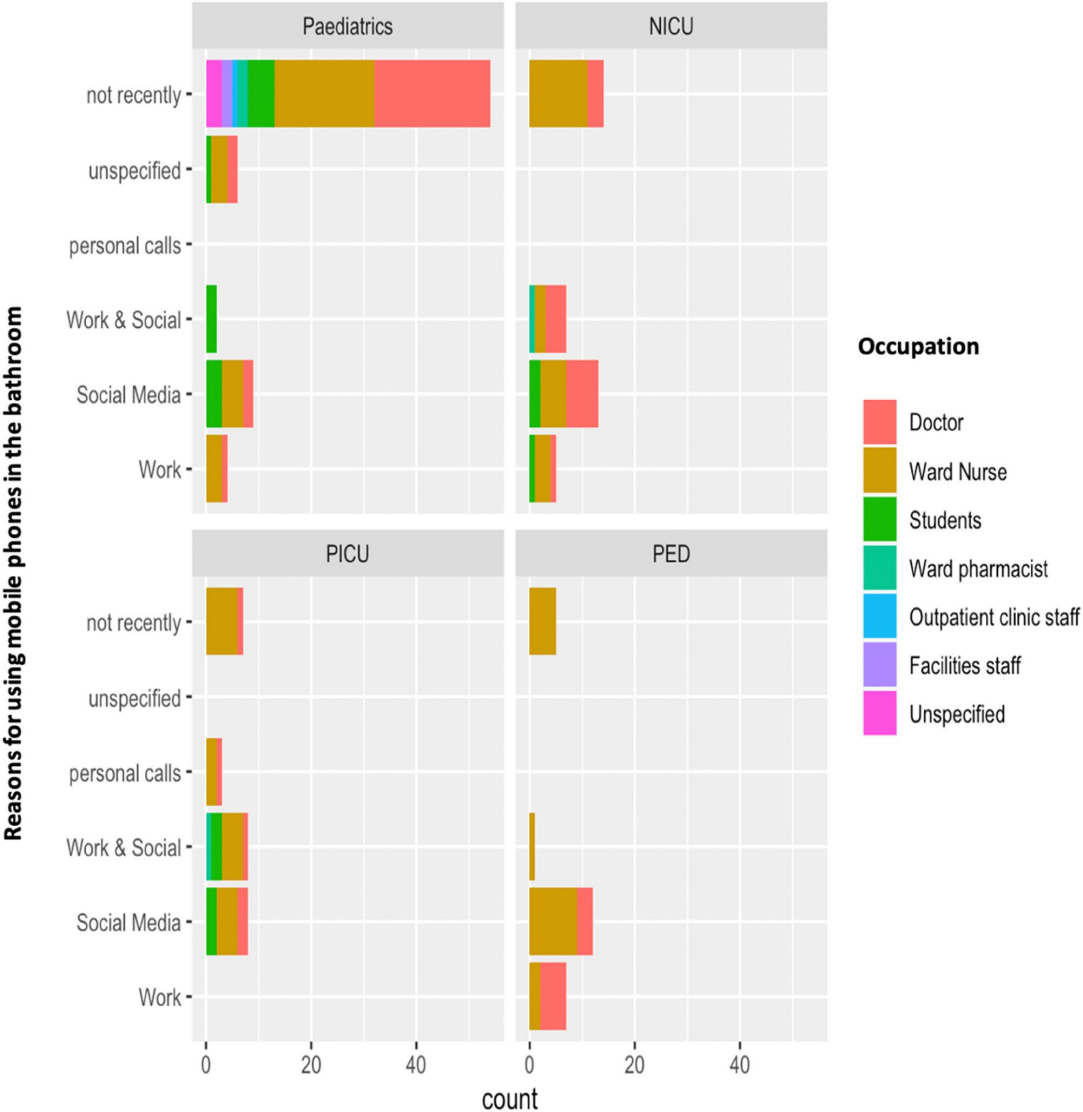
Mobile phone use in the bathroom and staff role across four different hospital wards (General Paediatrics, Neonatal Intensive Care Unit, Paediatric Intensive Care Unit, Paediatric Emergency Department).

Interestingly, there were 86 individuals who admitted to using their mobile device in the bathroom, whereas 79 individuals did not. This included a large number of doctors and ward nurses (Fig. 2). Approximately, 49% of ‘yes’ responders claimed to use their devices in the bathroom for social media, followed by 21% who answered, ‘work and social media’, 18.6% answered ‘work’, 8% were unspecified and 3.5% used their phones to answer personal phone calls (Fig. 2).

**Figure 3:**
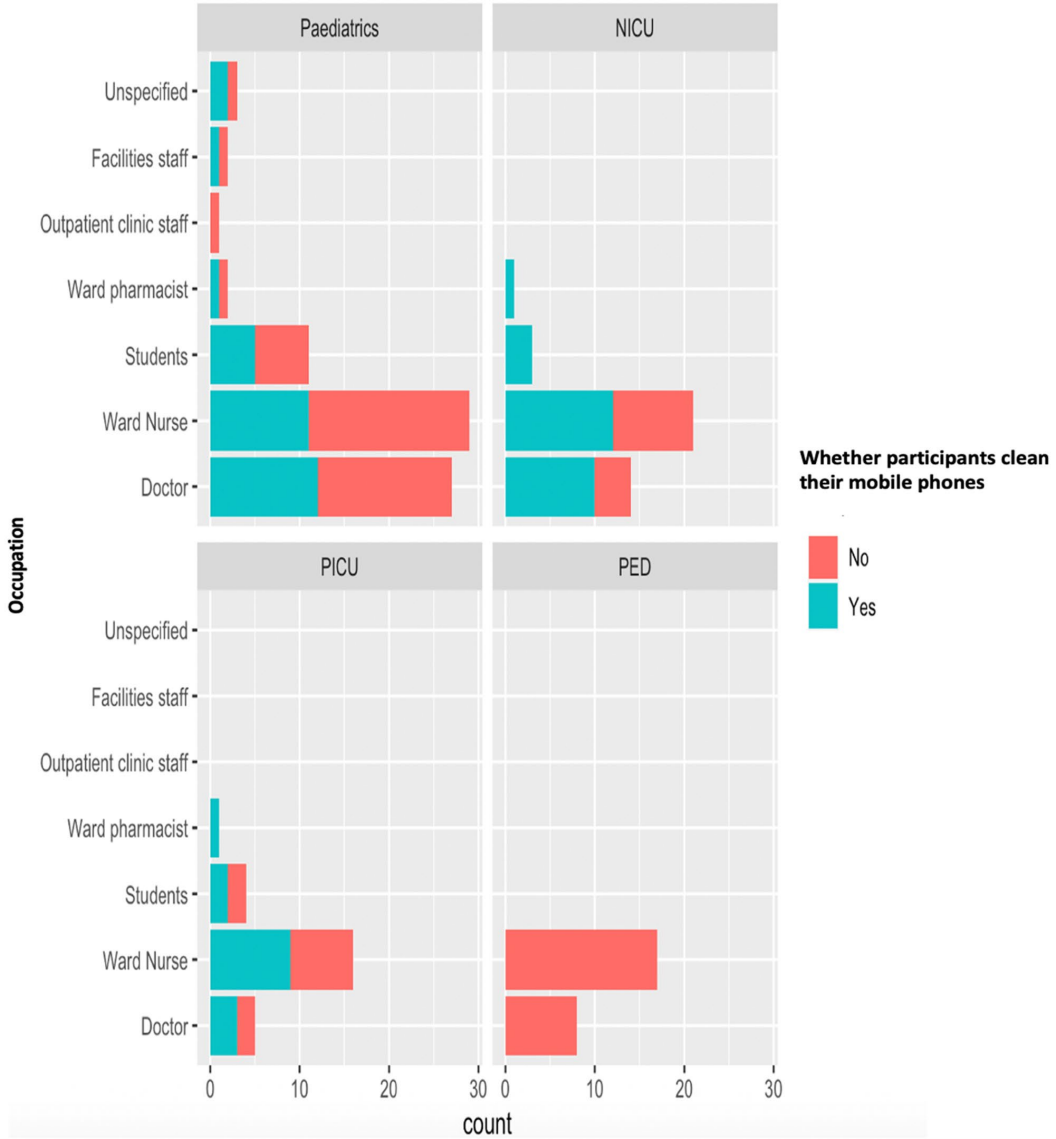
Mobile phone sanitisation and staff role across four different hospital wards (General Paediatrics, Neonatal Intensive Care Unit, Paediatric Intensive Care Unit, Paediatric Emergency Department).

When investigating whether participants have ever cleaned their mobile phones, 57% of respondents revealed that they had never cleaned their devices (Fig. 3). All respondents from the PED have never cleaned their mobile phones.

**Figure 4:**
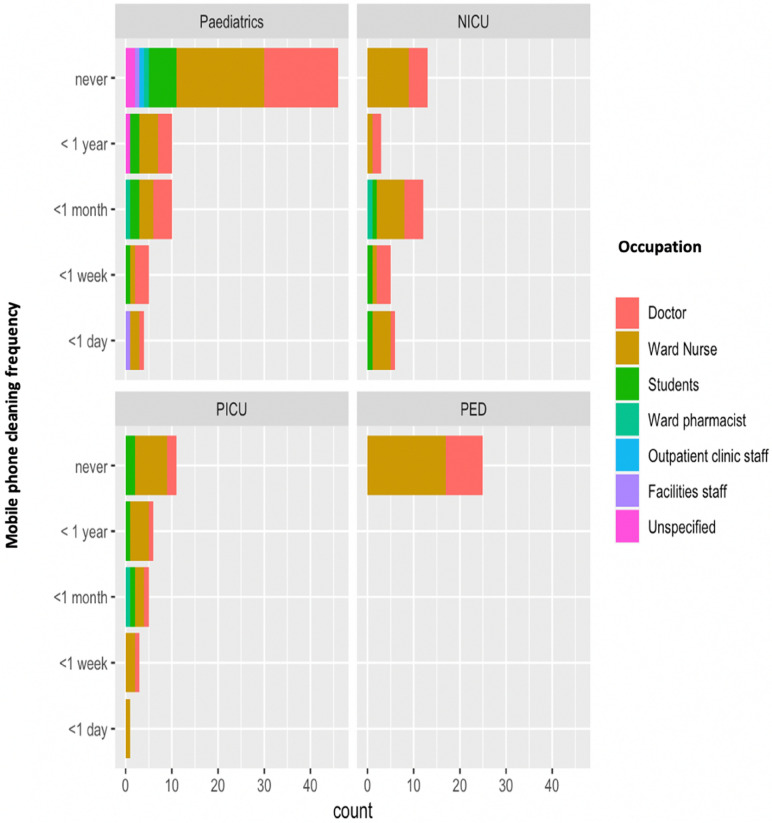
Mobile phone cleaning frequency and staff role across four different hospital wards (General Paediatrics, Neonatal Intensive Care Unit, Paediatric Intensive Care Unit, Paediatric Emergency Department).

In total, there were 92 responders who had never cleaned their mobile phone and 73 who had cleaned their phone at some timepoint. 38% of responders who have cleaned their phones, did so within the past month, 26.7% within the past year, 18.3% within the past week, 15.5% within the past day and 1.4% did not specify (Fig. 4).

**Figure 5:**
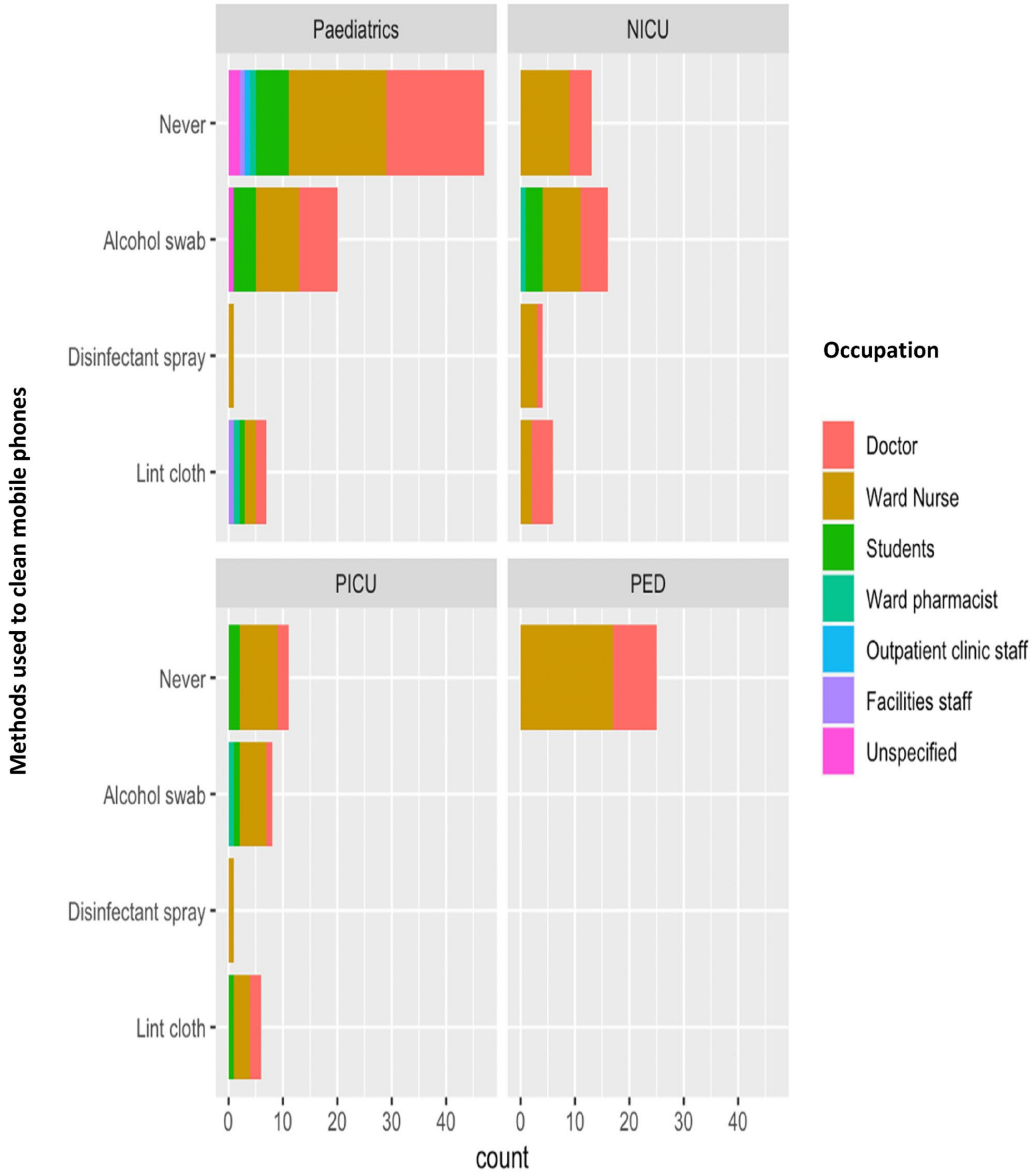
Mobile phone cleaning method and staff role across four different hospital wards (General Paediatrics, Neonatal Intensive Care Unit, Paediatric Intensive Care Unit, Paediatric Emergency Department).

When comparing different disinfection techniques, the most popular answer was an alcohol swab (63.7%), followed by a lint felt cloth (27.5%) and finally a disinfectant spray (8.7%) (Fig. 5).

**Figure 6:**
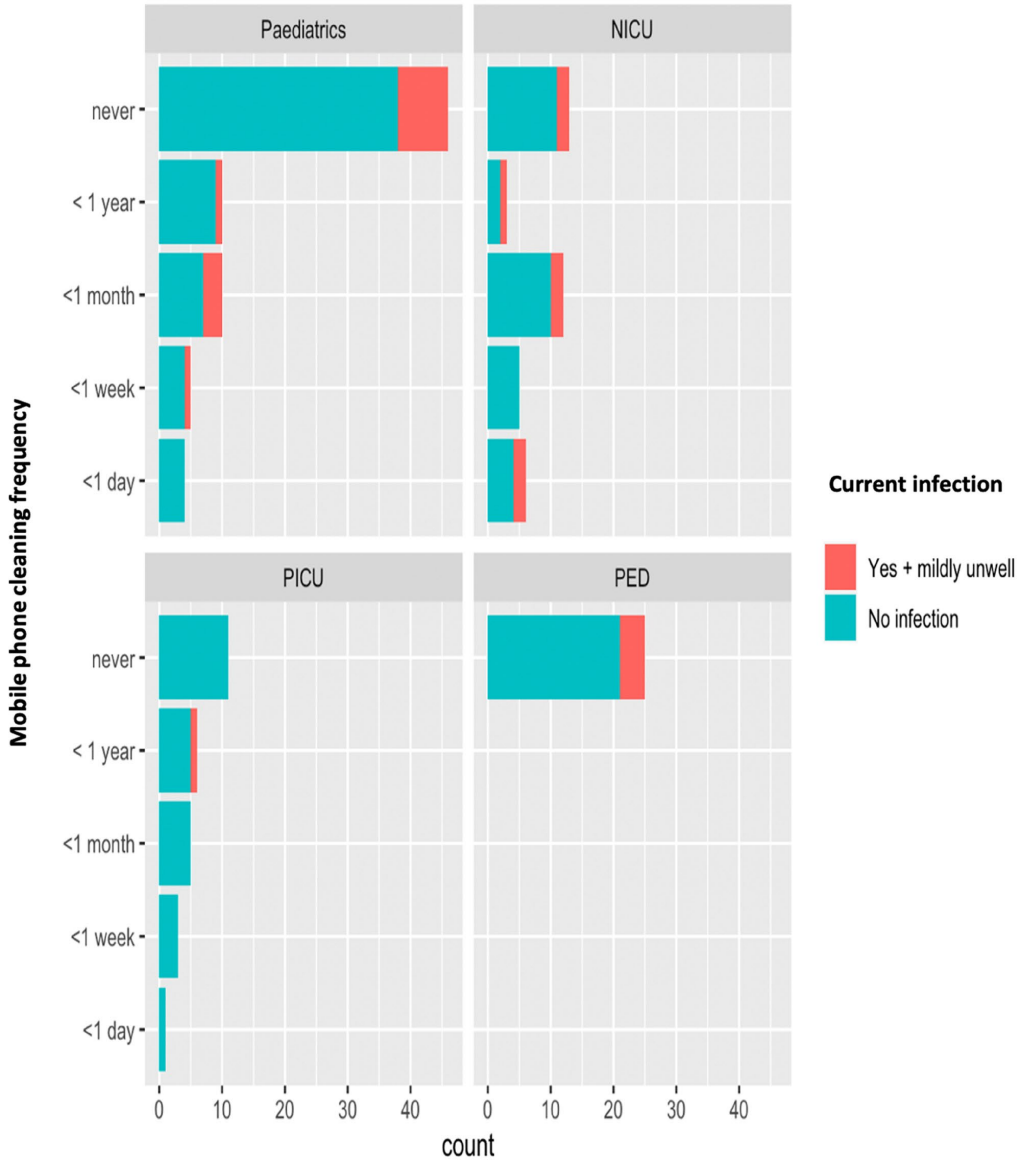
Comparison of mobile phone cleaning frequency and whether healthcare workers (medical staff and non-medical staff) self-reported to be suffering from an infection across four different hospital wards (General Paediatrics, Neonatal Intensive Care Unit, Paediatric Intensive Care Unit, Paediatric Emergency Department).

There were 16% (27/165) individuals who self-reported to be suffering from some kind of infection and feeling mildly unwell. Of those 27 individuals, 55% (15/27) self-reported to never have had their phones cleaned (Fig. 6) with the majority working in General Paediatrics and Paediatric Emergency Department.

### Chi-squared analyses

The Chi-Squared Test for Independence showed that occupation influences the hygiene habits of healthcare workers with respect to their mobile phone use in the bathroom (x^2^(2) = 21.53; P ≤ 0.01 **), in addition to the frequency with which their mobile phones are cleaned (x^2^(25) = 184.92; P ≤ 0.01 **), as outlined in Table 2.

**Table 2:** Factors associated with mobile phone hygiene and decontamination procedures.

	Age		Sex		Occupation	
	χ2 and (df)	P-value	χ2 and (df)	P-value	χ2 and (df)	P-value
Mobile phone use in Bathroom	6.42 (10)	0.77	14.62 (10)	0.14	21.53 (2)	< 0.01**
Frequency of cleaning	4.04 (10)	0.94	7.42 (10)	0.68	184.92 (25)	< 0.01**
Method of cleaning	4.39 (10)	0.92	3.80 (6)	0.70	8.57 (12)	0.73

Age and sex did not influence whether staff members used their devices in the bathroom or the frequency with which they cleaned their devices. Additionally, occupation did not influence the method with which staff members cleaned their mobile phones.

## Discussion

The results of this survey provide further evidence of the risk presented by mobile phones used by healthcare workers in hospital wards.

The majority of respondents have a large screen smartphone with large surface area for microbial contamination. A recent study demonstrated that viruses (specially SARS-CoV-2) are capable of surviving on glass surfaces (e.g., mobile phones) for extended periods of up to 28 days, in comparison to previous estimated survival times of 14 days^10^. This provides further evidence for the potential risk of microbial transmission that mobile phones represent.

In this study, no participants were currently taking antibiotics at the time of the survey, however 22% of doctors and 12% of ward nurses who reported to be feeling mildly unwell and perceived to be suffering from some kind of infection. Whilst clear guidelines for hand washing are already implemented in healthcare settings, the process might be ineffective as mobile phones, contaminated with microbes and used as essential tools at work, may re-infect hands and therefore lead to microbial spread and contamination in such settings.

Additionally, evidence of active microbial shedding from asymptomatic individuals is taking place and naturally occurring. In our 2020 scoping review, we hypothesised that SARS-CoV-2 infected individuals, symptomatic and asymptomatic, in the COVID-19 pandemic can potentially contaminate mobile phones with such devices probably contributing to the spread of the virus. With new research following our warning, a new study had illustrated the vital role that mobile phones play in the transmission of SARS-CoV-2 with such virus found on these surfaces up to 28 days^10^.

Of concern, the results of our survey showed that approximately 12% of ward nurses and 22% of doctors reported to be feeling mildly unwell while attending the workplace at the day of our study. Along with mobile phones fomites, this finding is exposing importantly the challenge public health authorities have to manage both epidemic viruses^11^, and pandemics like COVID-19. Recently, our team has demonstrated that mobile phone contamination poses a threat and that there is a lack of proper disinfection protocols and compliance^5^. In the literature, 70% isopropyl alcohol wipes are recommended to disinfect contaminated surfaces^12,13^, however, this is rarely followed as standard practice. In this study, of the 72 individuals who did clean their mobile phones, 27.5% (n = 19) did not used an appropriate technique, but instead used a lint felt cloth. Lint cloths are usually provided by manufacturers for cleaning of surfaces but not specifically for decontaminating the surface which is a general concern not only in healthcare settings but as well in the community. Additionally, unproper decontamination of mobile phones in healthcare setting might lead to the propagation of these mobile phone’s microbes in the community when healthcare staff finish their work^14^. According to the study of Brady et al., 50.9% of participants indicated that they have never cleaned their phone outside of the hospital environment^8^.

A key finding in this study was that whilst 98% of participants acknowledged and believed that their devices have the potential to harbour pathogenic microorganisms, relatively number of participants regularly cleaned their devices. Interestingly, the lack of phone hygiene also varied between hospital departments, with, 100% (n = 25) of the healthcare workers from the PED reporting to having never cleaned their phones. Participants from other wards including the NICU and PICU stated they did not claim to have cleaned their phones; however, they were still the minority. Of note, 73% and 27% of respondents utilised smartphone devices with large and small screens respectively. With the diversity of mobile phone size, larger devices will provide additional contaminable surface area.

Our survey showed that 52% of participants (n = 86) used their devices in the bathroom for various reasons, which further emphasises the unhygienic environments that mobile devices/smartphones are constantly being used within. Whilst all participants stated to wash their hands after using the bathroom, the ones using mobile phones in bathroom create the potential for microbial cross-contamination back to their hands. This further highlights the ‘Trojan Horse’ characteristics of mobile phones bypassing the gold standard hand washing practices in healthcare settings.

The use of mobile phone in toilets is of concern especially recently with the COVID-19 pandemic. Recent research has discovered SARS-CoV-2 virus in present in high amounts in faeces of infected individuals and subsequently wastewater^15^. The viral target ACE2 receptor present is present in the gastro-intestinal-tract and viral tropism is maintained for extended periods of time^16^. The use of mobile devices in the bathroom may contribute to the propagation of SARS-CoV-2 in the world. Of interest, a recent Chinese study investigating asymptomatic carriers of the SARS-CoV-2 virus in an isolation ward, showed that all carriers had positive anal swabs for the virus. Interestingly, among 96 different environmental sampling in this ward, only three samples were positive for the virus and included a cell phone, a cell phone shelf and a bedside rail^17^. Common fecal-derived microbes are frequently found on mobile phones such as *Acinetobacter*, *Enterococci* species^5^. In this scoping review, *E. coli* bacteria were identified on healthcare and community mobile phones in over a third of all studies investigated published in 24 different countries^5^.

Of note, when individuals are questioned about their hygiene habits, there is a chance that respondents will alter their current behaviour in response to their awareness of being observed. This is referred to as the Hawthorne effect^18^ and may have resulted in increased awareness of mobile phone contamination of microorganisms and may have prompted some respondents to report that they do clean their mobile phones.

## Conclusions

Mobile phones and smartphones are neglected contaminated platforms acting as ‘Trojan Horses’ for microbial in healthcare settings and may be partly contributing for the high occurrence of nosocomial diseases. Within the healthcare settings tested in this study, 87% of respondents are claiming that their device is an essential tool for their job with most staff (98%) believing that their devices harbour micro-organisms. Unexpectedly, 57% do not decontaminate their mobile phones frequently enough (weekly or more often), with most respondent using anyway inappropriate decontamination techniques. Along with the ubiquitous use of mobile phones in healthcare institutions, 52% of staff surveyed use their mobile phones in the bathroom. This habit may contribute further to a higher degree of microbial contamination on phones and might be responsible for cross-contamination back to their hands even with the practice of hand washing while exiting the bathroom. Finally, 16% of participants of this study self-reported to be suffering from some kind of infection with more than half reporting they never have had their phones cleaned. To conclude, mobile phones are fomites in healthcare settings and are neglected sources for the potential spread of microbes. Phone disinfection guidelines or regulations would likely reduce microbial phone contamination which may, in turn, reduce microbial cross-contamination to hands and potentially lead to lower nosocomial infections.

## Author’s recommendations

Research reporting on mobile phone microbial contamination has consistently reported a lack of proper protocols for phone disinfection in medical settings but also in public areas. Overall, this study provides further evidence for the need for medical (and public) health regulations on mobile phone and smartphone sanitisation. With 2020 research reporting SARS-CoV-2 virus present on phones for 28 days, this research provides further evidence for global public health authorities to advise all medical institutions to implement phone microbial decontamination protocols such as UVC disinfection techniques devices dedicated for phones. We urge for further scientific investigations to (1) expose further phones as fomites ‘Trojan Horse’ and (2) ensure worldwide health authorities actively and urgently implement regulations and policies to clean phones.
